# Reframing type 1 diabetes through the endocannabinoidome-microbiota axis: a systems biology perspective

**DOI:** 10.3389/fendo.2025.1576419

**Published:** 2025-05-29

**Authors:** Wojciech Łukowski

**Affiliations:** CARE FOR T1D, Bydgoszcz, Poland

**Keywords:** type 1 diabetes (T1D), endocannabinoid system (ECS), gut microbiota, autoimmune diseases, intestinal permeability, metabolic dysregulation, short-chain fatty acids, TRPV

## Abstract

Type 1 diabetes (T1D) has long been recognized as a T-cell-driven autoimmune disease. However, growing evidence highlights the involvement of metabolic, inflammatory, and gut microbiota-related factors in its progression. The endocannabinoid system (ECS), a key regulator of immune and metabolic homeostasis, has been increasingly implicated in autoimmune pathophysiology, particularly through its interactions with gut-derived metabolites. This hypothesis article underscores the need to reframe T1D pathophysiology by integrating ECS dysfunction, gut dysbiosis, and metabolic imbalances into a systems biology framework. The proposed Endocannabinoidome-Microbiota (ECBoM) model highlights a shared hallmark of autoimmunity—SCFA depletion, increased intestinal permeability, and ECS dysregulation—as key drivers of chronic inflammation and immune dysfunction. These disturbances, observed in T1D as well as in celiac disease, Hashimoto’s thyroiditis, rheumatoid arthritis, and multiple sclerosis, suggest a common immune-metabolic axis across autoimmune disorders. Recognizing ECS dysregulation as a systemic feature of autoimmunity opens avenues for novel therapeutic interventions, including ECS-targeted treatments, microbiota modulation, and phytocannabinoid-based therapies. This article highlights the necessity of conducting large-scale, multi-omics studies to establish disease-specific ECS signatures, linking endocannabinoid profiling, microbiota composition, and metabolic biomarkers to disease progression. By advocating for a paradigm shift in T1D research, this article emphasizes the importance of exploring new mechanistic references to develop targeted, immune-metabolic interventions that could reshape treatment strategies and improve clinical outcomes in T1D and related autoimmune diseases.

## Introduction

Type 1 diabetes (T1D) is a multifactorial autoimmune disease arising from a complex interplay between genetic susceptibility, environmental factors, and immune dysregulation (1). While considerable progress has been made in elucidating the genetic and immunological underpinnings of T1D, current models fail to fully explain the mechanisms initiating the breakdown of tolerance toward pancreatic β-cells (1, 2). In recent years, growing attention has focused on the endocannabinoid system (ECS) and gut microbiota as two dynamic, interconnected regulators of immune and metabolic homeostasis (3, 4).

The ECS is an evolutionarily conserved lipid-based signaling system composed of endocannabinoids such as anandamide (AEA) and 2-arachidonoylglycerol (2-AG), their receptors (CB1, CB2, TRPV1), and associated metabolic enzymes (FAAH, MAGL, DAGL) (5, 6). Unlike classical neurotransmitters or hormones, endocannabinoids are synthesized on demand, locally, in response to cellular stress or inflammatory stimuli (7). This system acts as a local buffer, fine-tuning immune responses, intestinal barrier integrity, and cellular stress adaptation (8, 9).

Emerging evidence indicates a bidirectional relationship between ECS signaling and gut microbiota composition, forming a regulatory axis known as the endocannabinoidome–microbiota axis (ECBoM) (3, 4). Gut dysbiosis has been shown to disrupt ECS tone through reduced production of short-chain fatty acids (SCFAs) and increased translocation of microbial-derived lipopolysaccharides (LPS), leading to chronic ECS overstimulation (10–12). This dysregulation, in turn, impairs intestinal barrier integrity, promotes systemic inflammation, and affects immune tolerance (9, 11, 13).

**Figure 1:**
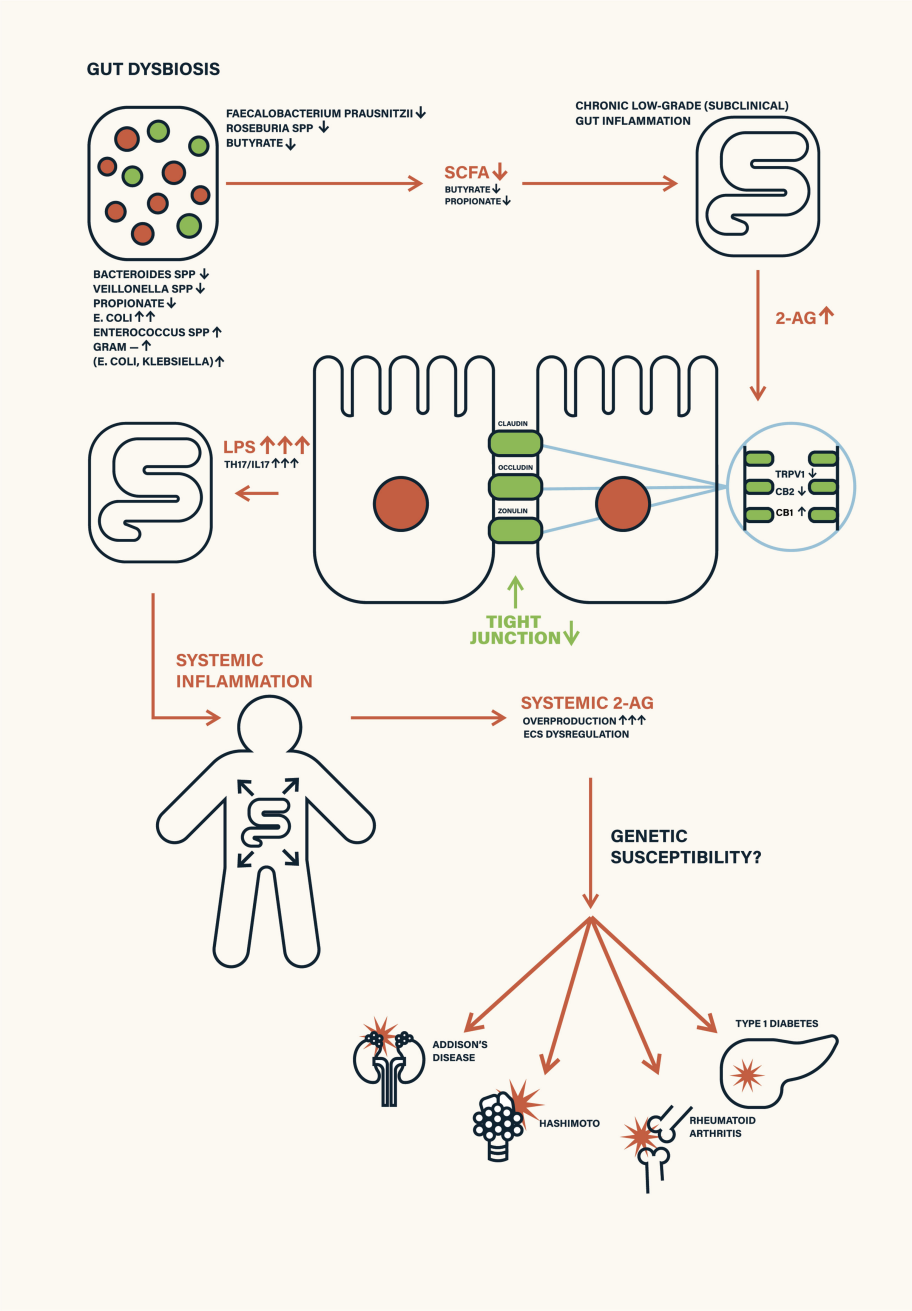
Schematic depiction of environmental triggers—such as antibiotic exposure, poor diet, infant formula feeding, and stress—disrupting gut microbiota and endocannabinoid system (ECS) homeostasis. This leads to ECS receptor desensitization, increased intestinal permeability (leaky gut), and systemic inflammation, ultimately contributing to autoimmune disease manifestation.

In this work, we propose a novel sequential model of T1D pathogenesis, grounded in molecular ECS-microbiota interactions, that links gut dysbiosis to β-cell autoimmunity, as pictured in the **Figure 1**. This model outlines a stepwise cascade:

Gut dysbiosis → SCFA depletion (4, 10)→ compensatory 2-AG overproduction → ECS overstimulation (3, 14)→ CB2 and TRPV1 receptor desensitization (15–18) → increased intestinal permeability (“leaky gut”) (11, 13)→ systemic inflammation (19)→ immune ECS dysregulation due to excess 2-AG → disruption of the blood–pancreas barrier by ECS dysregulation → infiltration of inflammatory mediators into pancreatic islets → ECS dysregulation in pancreatic β-cells → closure of GDDC and VDCC calcium channels → impaired calcium influx into β-cells → suppression of insulin secretion → activation of β-cell apoptotic pathways → autoimmune targeting and destruction of β-cells (1, 20, 21).

This mechanistic framework integrates existing findings on ECS and microbiota into a coherent, testable hypothesis that may explain the early molecular events triggering T1D in genetically predisposed individuals. Furthermore, it highlights new therapeutic and preventive avenues by targeting ECS-microbiota balance to maintain immunometabolic homeostasis.

## Endocannabinoid system and microbiota interactions

The endocannabinoid system (ECS) is an evolutionarily conserved lipid-signaling system extensively involved in maintaining homeostasis across multiple physiological processes, including metabolism, inflammation, immunity, and gut function (5, 22). Unlike classical neurotransmitters or circulating hormones, endocannabinoids such as anandamide (AEA) and 2-arachidonoylglycerol (2-AG) are synthesized *on demand* at the site of need, in response to local cellular stress, inflammatory stimuli, or membrane depolarization (7, 14). This localized and transient mode of synthesis underscores their role as fine-tuned regulators of homeostasis rather than long-range systemic signals. The ECS comprises endocannabinoids (primarily anandamide [AEA] and 2-arachidonoylglycerol [2-AG]), their associated receptors (CB1, CB2, TRPV1, GPR55), and metabolic enzymes such as fatty acid amide hydrolase (FAAH), monoacylglycerol lipase (MAGL), and diacylglycerol lipase (DAGL) (6, 22). Recent studies suggest that ECS signaling intricately interacts with gut microbiota, forming a critical axis termed the endocannabinoidome-microbiota axis (ECBoM). This interaction modulates gut barrier integrity, intestinal inflammation, immune responses, and systemic metabolic homeostasis, positioning ECBoM dysregulation as a potential pivotal event in autoimmune disorders, notably type 1 diabetes (T1D) (1, 20).

Building on these observations, we propose a sequential model of pathogenesis that mechanistically links gut dysbiosis to autoimmune β-cell destruction in T1D through progressive disruption of ECS-mediated homeostasis: This dysregulation is hypothesized to follow a specific, sequential cascade (1, 3, 7):

intestinal dysbiosis (3)→ reduction in SCFA production (3)→ compensatory overproduction of 2-AG (14) → chronic (often subclinical) intestinal inflammation (9)→ ECS dysregulation in the gut (12)→ tight junction (TJ) downregulation → leaky gut (13)→ translocation of pro-inflammatory mediators into systemic circulation (19)→ generalized systemic inflammation (1) → immune system–driven overproduction of 2-AG (15, 16)→ ECS dysregulation in immune cells (8) → 2-AG-induced dysfunction of the blood–pancreas barrier (depending on ECS-related genetic susceptibility) (23)→ penetration of inflammatory cytokines into pancreatic tissue (24)→ ECS dysregulation within pancreatic islets (20) → mitochondrial dysfunction and β-cell apoptosis signaling (25)→ immune recognition and targeting of β-cells (1)→ clinical onset of type 1 diabetes.

The human gut microbiota consists of trillions of microorganisms collectively contributing to essential host functions such as digestion, metabolism of dietary components, synthesis of critical nutrients, and modulation of immune function (10, 26). Dysbiosis, defined as a perturbation in the composition and diversity of gut microbiota, is increasingly recognized as a major factor contributing to autoimmune and inflammatory disorders, including T1D (2, 27, 28). Notably, dysbiotic conditions frequently correlate with altered ECS activity and expression, suggesting a complex bidirectional interaction between ECS and microbiota (3, 4, 12).

Beyond the classical endocannabinoids anandamide (AEA) and 2-arachidonoylglycerol (2-AG), the ECS also encompasses a broader family of lipid mediators often referred to as “endocannabinoid-like compounds”, including oleoylethanolamide (OEA) and palmitoylethanolamide (PEA). These molecules do not bind strongly to CB1 or CB2 receptors but exert profound anti-inflammatory and immunoregulatory effects via activation of PPAR-α (14, 22). Notably, PEA has demonstrated protective effects in models of neuroinflammation and mast cell regulation (29), while OEA contributes to metabolic homeostasis, satiety regulation, and gut signaling (3). Their actions complement canonical ECS pathways, adding another layer of complexity to ECS–microbiota–immune interactions, and their inclusion in future studies may provide a more comprehensive understanding of endocannabinoid tone in T1D pathogenesis.

Importantly, the ECBoM axis cannot be fully understood without considering the enzymatic regulation of endocannabinoid tone. Diacylglycerol lipase (DAGL), responsible for 2-AG synthesis, and monoacylglycerol lipase (MAGL), responsible for its degradation, are dynamically regulated by both inflammatory cytokines and microbial metabolites. For example, lipopolysaccharides (LPS) can upregulate DAGL expression in immune cells, enhancing 2-AG synthesis and promoting ECS overstimulation (3, 4). Simultaneously, SCFAs like butyrate may downregulate DAGL activity and support MAGL function, thus restoring ECS balance (10). These reciprocal interactions suggest that microbiota-derived signals may fine-tune ECS activity not only via receptor modulation but also through enzymatic control of endocannabinoid turnover. This enzymatic layer represents a critical regulatory node that warrants further exploration in future studies.

Gut microbiota directly influence ECS signaling through several distinct molecular mechanisms. A central mediator of this interaction is the production of short-chain fatty acids (SCFAs), primarily acetate, propionate, and butyrate, synthesized through bacterial fermentation of dietary fibers. SCFAs actively regulate ECS signaling by modulating the expression of ECS components in gut epithelial and immune cells. In particular, SCFAs have been shown to upregulate CB2 receptor expression on intestinal epithelial cells and immune cells, reinforcing intestinal barrier function and promoting anti-inflammatory responses (3, 25). Simultaneously, SCFAs enhance epithelial tight junction proteins, thereby decreasing gut permeability and limiting translocation of inflammatory mediators, pathogens, or bacterial components such as lipopolysaccharide (LPS) into systemic circulation (10, 11).

Conversely, a dysbiotic microbiome characterized by a reduction of beneficial SCFA-producing bacteria (e.g., *Faecalibacterium prausnitzii*, *Roseburia intestinalis*) is associated with decreased SCFA levels, impaired intestinal epithelial integrity, increased gut permeability, and consequent systemic inflammation (10, 28). Under dysbiotic conditions, elevated intestinal permeability allows increased translocation of microbial-derived LPS, which in turn activates toll-like receptor 4 (TLR4) signaling pathways on immune cells, leading to sustained ECS activation (3, 9). Specifically, increased systemic LPS directly elevates 2-AG levels via induction of DAGL activity, subsequently overstimulating ECS receptors, primarily CB1 and TRPV1 (6, 12, 18). Importantly, it is the resulting dysregulation of intestinal ECS – marked by CB2/TRPV1 desensitization and CB1 overactivation – that directly weakens tight junction integrity and leads to the structural collapse of the epithelial barrier (17, 25).

Prolonged ECS receptor overstimulation due to persistent dysbiotic signals (notably elevated LPS and diminished SCFAs) leads to receptor desensitization, particularly within TRPV1-rich tissues such as intestinal epithelia and pancreatic β-cells (17, 18, 25). Chronic 2-AG elevation results in TRPV1 channel desensitization, profoundly disturbing intracellular calcium homeostasis, mitochondrial functions, and cellular survival signaling pathways, thereby predisposing cells to inflammatory damage and apoptosis (15, 16). Thus, gut microbiota dysbiosis indirectly contributes to tissue-specific vulnerability through ECS receptor dysregulation, further amplifying systemic inflammation and immune dysregulation (1, 8).

Moreover, dysbiosis-induced changes in intestinal permeability and ECS signaling dramatically affect intestinal immune cell populations. The gut mucosal immune system, a primary site for immune tolerance and inflammatory control, expresses abundant ECS receptors, predominantly CB2, that regulate local inflammatory responses and T-cell differentiation (8, 25). CB2 signaling notably promotes the generation and maintenance of regulatory T-cells (Tregs) while suppressing inflammatory Th17 cell responses (29). Under healthy microbiota conditions, robust CB2 receptor signaling induced by SCFA-rich environments favors immune tolerance and intestinal barrier integrity (3, 4). Dysbiotic conditions disrupt this regulatory axis, leading to diminished CB2 expression, reduced Treg generation, and increased Th17-driven inflammatory responses, profoundly shifting intestinal immune homeostasis toward a pro-inflammatory phenotype, which systemically predisposes to autoimmunity (1, 12).

The ECS reciprocally modulates microbiota composition, demonstrating bidirectional interaction. ECS signaling pathways directly regulate intestinal motility, secretion, barrier function, and antimicrobial peptide production, significantly shaping microbial populations in the gut (3, 4). Animal model studies demonstrate that genetic deletion or pharmacological blockade of CB1 receptors alters intestinal motility and secretory functions, resulting in significant shifts in microbiota composition characterized by increased pro-inflammatory bacteria and reduced SCFA-producing populations (9, 12). Similarly, CB2 receptor knockout mice exhibit increased gut inflammation and altered microbiota profiles, reflecting ECS’s essential role in maintaining intestinal immunological and microbial homeostasis (8, 25).

Clinical and preclinical observations highlight that conditions characterized by ECS dysregulation, such as obesity, inflammatory bowel disease, or metabolic syndrome, consistently associate with microbiota dysbiosis, supporting ECBoM’s central role in human disease (4, 9, 22). Importantly, emerging clinical data from T1D patients reveal notable alterations in ECS tone, particularly elevated circulating 2-AG levels accompanied by intestinal microbiota shifts indicative of reduced SCFA-producing bacteria and increased LPS-bearing Gram-negative bacteria (10, 30). These observations suggest that ECBoM axis dysregulation might actively contribute to T1D pathogenesis rather than merely being secondary to the autoimmune disease process (1).

Given ECBoM’s critical immunometabolic regulatory roles, therapeutic modulation of ECS signaling or gut microbiota represents an intriguing strategy for T1D management (1, 3). Potential interventions include probiotics enriched with SCFA-producing bacteria, dietary strategies aiming at microbiota restoration, and pharmacological ECS modulation (e.g., CB2 agonists, selective TRPV1 modulators, inhibitors of 2-AG metabolism), or phytocannabinoids (7, 25, 31, 32). Although promising, these approaches require rigorous clinical validation and consideration of individualized microbiota profiles and ECS states, emphasizing the complexity of clinical translation (3, 10).

Future research directions should prioritize detailed characterization of ECBoM interactions through multi-omics approaches, including metagenomics, metabolomics, transcriptomics, and proteomics, to comprehensively define microbiota-driven ECS alterations and their consequences for immune homeostasis and metabolic regulation (3, 4, 12). Additionally, longitudinal clinical studies of genetically predisposed individuals may reveal ECBoM biomarkers predictive of T1D onset, enabling early preventive interventions targeting ECS restoration and microbiota rebalancing (33–35).

In conclusion, ECS and gut microbiota interactions constitute a critical homeostatic regulatory axis whose dysregulation significantly contributes to T1D pathogenesis (1, 3). Detailed understanding of ECBoM’s molecular interactions and pathophysiological mechanisms provides a compelling framework for novel preventive and therapeutic strategies, marking a potential paradigm shift in the management of autoimmune diabetes (4, 12, 31).

## Genetic susceptibility: ECS-related genes and interactions

Type 1 diabetes (T1D) is widely recognized as a genetically complex autoimmune disease, with a strong influence exerted by the human leukocyte antigen (HLA) system within the major histocompatibility complex (MHC) on chromosome 6. The specific HLA haplotypes, notably DR3-DQ2 and DR4-DQ8, significantly increase T1D susceptibility, collectively accounting for approximately half of the genetic risk associated with disease development (19). These HLA molecules play pivotal roles in antigen presentation and T-cell activation, fueling autoimmune responses targeting pancreatic β-cells. Beyond HLA, numerous non-HLA genes, including PTPN22, CTLA4, IL2RA, FOXP3, and INS, further modulate immune responses, influencing T-cell activation, immune tolerance, and cytokine regulation, contributing cumulatively to genetic predisposition (33, 34). Yet, these known genetic loci explain only a fraction of the observed T1D heritability, underscoring the existence of additional susceptibility genes involved in metabolic and inflammatory regulation, particularly within newly explored regulatory systems, such as the endocannabinoid system (ECS) (1, 3).

The ECS consists of endocannabinoids (e.g., anandamide and 2-arachidonoylglycerol [2-AG]), cannabinoid receptors (CB1, CB2), transient receptor potential vanilloid channels (TRPV1), and enzymes responsible for endocannabinoid metabolism (FAAH, MAGL, DAGL). Recent evidence indicates that genetic variations in ECS components may profoundly influence susceptibility to autoimmune conditions, including T1D (1, 3, 25). ECS genes harbor functional polymorphisms that can critically modulate receptor expression levels, ligand availability, downstream signaling efficiency, and overall immunoregulatory capacities, thus significantly impacting autoimmune disease susceptibility (16, 36).

Genetic variations in the CB2 receptor gene (CNR2) hold particular relevance due to CB2’s essential immunoregulatory functions. CB2 receptors, primarily expressed on immune cells, control inflammatory processes by suppressing pro-inflammatory cytokine release and promoting regulatory T-cell (Treg) stability. Polymorphisms such as rs35761398 and rs2501432 in CNR2 markedly reduce receptor expression and signaling efficacy (36), diminishing CB2’s anti-inflammatory capabilities. The resultant immunoregulatory impairment predisposes carriers of these variants to increased inflammation and autoimmunity, notably by compromising Treg-mediated peripheral tolerance (16). Consequently, individuals harboring CNR2 risk alleles face an elevated risk of autoimmune destruction of pancreatic β-cells, which may accelerate T1D onset and severity (1).

Similarly, genetic variants in the CB1 receptor gene (CNR1), exemplified by rs1049353, modulate receptor activity and alter metabolic and inflammatory signaling cascades (6, 22). Dysregulated CB1 signaling contributes significantly to metabolic inflammation, insulin resistance, and cytokine dysregulation, thereby creating a persistent inflammatory milieu conducive to autoimmune activation (31). Overactivation of inflammatory pathways through abnormal CB1 signaling promotes autoreactive T-cell activation and β-cell stress, thereby increasing the susceptibility to T1D progression, especially in genetically predisposed individuals (1).

Moreover, polymorphisms within TRPV1 gene channels further compound genetic susceptibility. TRPV1 channels are critical calcium-permeable cation channels abundantly expressed in pancreatic β-cells, intestinal epithelial cells, and immune cells (17, 18, 37). Specific variants, such as rs8065080, alter TRPV1 receptor sensitivity and channel gating properties, profoundly affecting cellular calcium homeostasis (17, 18). Disturbed calcium influx resulting from TRPV1 dysfunction enhances endoplasmic reticulum stress, mitochondrial dysfunction, and apoptosis in pancreatic β-cells, undermining cellular integrity and increasing vulnerability to autoimmune destruction (15, 24). Furthermore, impaired TRPV1 activity in intestinal epithelial cells compromises barrier integrity, amplifying intestinal permeability and exacerbating systemic inflammation, thus connecting genetic susceptibility directly to dysregulated ECS-mediated barrier functions and immune dysregulation (17, 18).

Enzymatic components of the ECS, such as FAAH and MAGL, also harbor influential genetic polymorphisms that significantly modify endocannabinoid tone by regulating anandamide and 2-AG metabolism. For instance, FAAH polymorphism rs324420 (C385A) results in reduced enzymatic activity, elevating systemic anandamide levels and disturbing immune homeostasis (14, 38). Elevated endocannabinoid levels disrupt normal immune regulatory networks, skew cytokine production toward pro-inflammatory phenotypes, and impair Treg differentiation, thereby heightening autoimmune susceptibility in genetically predisposed individuals (16).

Importantly, ECS-related gene polymorphisms do not act in isolation; rather, they intricately interact epistatically with classical autoimmune genetic factors, amplifying autoimmune susceptibility. Notably, interactions between CB2 receptor variants (CNR2) and the FOXP3 gene, critical for Treg cell differentiation and stability, have significant consequences (16, 25). Impaired CB2 signaling coupled with FOXP3 risk variants synergistically reduces Treg-mediated immune regulation, dramatically lowering thresholds required for autoimmune disease initiation. Similarly, epistatic interactions between CB1 receptor polymorphisms (CNR1) and PTPN22, a gene influencing T-cell receptor signaling, exacerbate autoreactive T-cell activation, inflammatory cytokine release, and metabolic dysregulation (1), further enhancing autoimmune vulnerability. TRPV1 variants interacting with polymorphisms in the IL2RA gene disrupt IL-2 signaling crucial for Treg stability and function, tipping immune balance toward pro-inflammatory Th17 dominance, thereby intensifying autoimmune pathology and pancreatic β-cell destruction (16, 17).

Collectively, ECS gene polymorphisms appear to modulate autoimmune susceptibility by acting as genetic integrators or “shock absorbers,” buffering genetic predispositions to autoimmunity under normal physiological conditions (25, 39). Healthy ECS functionality preserves immune homeostasis, reduces inflammation, and maintains metabolic stability, counteracting genetic predispositions toward autoimmune responses (1). However, sustained environmental triggers, notably gut dysbiosis (3, 16) and chronic inflammation, overwhelm ECS protective capacities, causing receptor desensitization and ECS dysfunction. This critical transition point removes ECS-mediated protective buffering, fully unveiling autoimmune genetic predispositions and precipitating clinical autoimmune diabetes (36).

The ECS thus represents a vital integrative genetic node, bridging classical autoimmune susceptibility genes with metabolic and inflammatory regulation (22, 31). Therapeutically targeting ECS signaling through pharmacological modulation of receptor activity or endocannabinoid metabolism could potentially restore ECS functionality, thus reestablishing immune homeostasis and significantly reducing autoimmune disease risk in genetically susceptible populations (40, 41). Further research elucidating precise molecular mechanisms underlying these genetic interactions and ECS functionality is critical for developing effective preventive strategies and targeted interventions in T1D and other autoimmune diseases (29, 36).

While ECS-related genetic polymorphisms alone may not be sufficient to initiate autoimmunity, they appear to function as amplifiers of risk in the context of environmental or microbial challenges (12, 35). For instance, carriers of both CNR2 and TRPV1 variants who experience early-life dysbiosis or chronic low-grade inflammation may cross a biological threshold of ECS dysfunction more rapidly than non-carriers (29, 36). This combinatorial effect between genotype and environment aligns with the “two-hit hypothesis” (19) and suggests that ECS polymorphisms may serve as predictive markers for personalized risk stratification in T1D and related autoimmune diseases (42).

## Environmental triggers, gut dysbiosis, and ECS dysfunction

Despite a significant genetic predisposition underlying type 1 diabetes (T1D), environmental factors are essential in triggering the manifestation of clinical autoimmunity, effectively translating latent genetic risk into overt disease (34, 43). The interplay between genetic susceptibility and environmental stimuli is particularly evident within the proposed endocannabinoidome-microbiota (ECBoM) framework (3, 4). Environmental triggers operate primarily by perturbing gut microbiota composition and diversity, initiating a cascade of microbial imbalance termed dysbiosis (2, 28). Gut dysbiosis subsequently disrupts endocannabinoid system (ECS) homeostasis, facilitating autoimmune activation through receptor overstimulation, chronic inflammation, barrier dysfunction, and impaired immune regulation (8, 12, 42).

The human gut microbiome is a dynamic and delicately balanced ecosystem sensitive to external perturbations. Factors pervasive in modern lifestyles—such as inappropriate antibiotic usage, chronic psychological stress, dietary patterns high in processed foods and refined sugars, cesarean deliveries, and early-life exposure to infant formulas—profoundly alter gut microbiota structure and function (28, 33). Antibiotics, especially during critical developmental windows, significantly reduce microbial diversity, selectively eliminating beneficial SCFA-producing bacterial species (e.g., *Faecalibacterium prausnitzii*, *Roseburia intestinalis*) and facilitating the proliferation of opportunistic pathogens (30, 44). Such disturbances limit the production of protective metabolites like short-chain fatty acids (SCFAs), crucial mediators of intestinal barrier integrity, immune tolerance, and ECS regulation (3, 4, 10).

Similarly, dietary factors typical of Western diets—characterized by excessive intake of saturated fats, refined carbohydrates, and artificial additives—promote microbial shifts toward pro-inflammatory bacterial populations (4, 10). These dietary habits enhance intestinal permeability (“leaky gut”) by downregulating epithelial tight junction proteins via reduced SCFA levels and increased inflammatory mediators (11, 13). Consequently, disrupted intestinal barriers permit translocation of microbial-derived endotoxins (lipopolysaccharides, LPS), dietary antigens, and other immunostimulatory compounds into systemic circulation, initiating sustained low-grade systemic inflammation (43, 45). Furthermore, early-life nutritional exposures, particularly the replacement of human breast milk with artificial infant formulas, profoundly influence gut microbiome colonization. Breast milk provides bioactive components, including human milk oligosaccharides (HMOs), immunoglobulins, and essential fatty acids, that promote microbiota maturation and ECS homeostasis (19, 34). Formula feeding, devoid of these natural microbiota-regulating components, predisposes infants to gut dysbiosis, potentially heightening autoimmune risk later in life (28, 30).

Chronic psychological stress, increasingly prevalent in modern society, also significantly modulates gut microbiota composition and diversity through neuroendocrine pathways, particularly the hypothalamic-pituitary-adrenal (HPA) axis (13). Stress-induced cortisol elevations directly influence gut microbial populations, reducing beneficial species and promoting inflammatory microbiota profiles (10, 11). Consequently, chronic stress results in microbiota-driven ECS dysregulation, amplifying intestinal permeability, inflammation, and autoimmune susceptibility (3, 4). Notably, modern societal lifestyles characterized by high stress, poor dietary habits, and frequent antibiotic use converge to systematically erode microbiome resilience, creating a milieu highly conducive to ECS dysregulation and subsequent autoimmune disease initiation in genetically predisposed individuals (30, 34).

These microbiome disruptions directly influence ECS signaling, initiating a maladaptive cycle of chronic ECS overstimulation, particularly through elevated 2-arachidonoylglycerol (2-AG) levels (3). Increased systemic exposure to microbial-derived endotoxins, such as LPS, induces ECS hyperactivity primarily via the enhanced expression and activity of diacylglycerol lipase (DAGL), responsible for 2-AG synthesis (4, 23). Sustained elevation of 2-AG leads to profound ECS receptor overstimulation, notably of TRPV1 and CB1, triggering receptor desensitization and reduced signaling efficacy (6, 17, 18). As previously detailed, rapid TRPV1 desensitization critically disrupts calcium homeostasis, mitochondrial function, and cellular integrity within tissues highly relevant to T1D pathogenesis, such as pancreatic β-cells and intestinal epithelium (15, 17, 24).

This receptor-level dysfunction, secondary to persistent environmental triggers, removes the protective buffering capacity normally conferred by ECS signaling (46). Under healthy conditions, ECS effectively mitigates inflammatory stressors, preserves intestinal barrier integrity, and maintains immune tolerance, thus preventing genetic susceptibility from translating into clinical disease (7, 8, 42). However, persistent ECS dysfunction resulting from chronic microbiota dysbiosis undermines these protective capacities, leading to sustained immune activation and loss of peripheral tolerance, thereby precipitating overt autoimmune diabetes (3, 45).

Crucially, the interaction between environmental triggers, gut dysbiosis, and ECS dysfunction exemplifies the concept of gene-environment interaction within the ECBoM framework (3, 4). Individuals genetically predisposed to T1D—particularly carriers of HLA risk alleles and ECS-related genetic variants (e.g., CNR2, TRPV1)—are inherently more vulnerable to environmental-induced microbiota perturbations and subsequent ECS dysfunction (29, 36). For these individuals, relatively modest environmental insults, commonplace in modern lifestyles, may suffice to initiate dysbiosis, ECS dysregulation, and progression toward clinical autoimmunity. Conversely, in genetically resilient individuals, identical environmental exposures might not elicit ECS dysfunction or overt autoimmune manifestations, highlighting the critical integrative role played by ECS genetics and microbiota composition in determining individual autoimmune susceptibility (3, 45).

Given this integrative framework, preventive strategies focused on minimizing environmental triggers and restoring microbiota-ECS homeostasis are emerging as attractive interventions to mitigate autoimmune risk (3, 4). Interventions such as dietary modifications emphasizing microbiota-supportive nutrients (e.g., prebiotics, probiotics, dietary fibers, omega-3 fatty acids) (10), phytocannabinoids (46), reduction in unnecessary antibiotic prescriptions (28), stress management techniques, and promotion of breastfeeding (44) could significantly reduce microbiota dysbiosis, ECS overstimulation, and subsequent autoimmune risk (26). These approaches, though intuitive and scientifically grounded, demand extensive clinical validation (3). A comparative overview of microbiota alterations, SCFA depletion, and barrier dysfunction across autoimmune diseases is provided in (**Table 1**). Nonetheless, the inherent plasticity of gut microbiota and ECS signaling pathways provides hope that proactive lifestyle interventions, particularly during early life and in genetically at-risk populations, could markedly alter autoimmune trajectories (45).

**Table 1:** Alterations in gut microbiota and SCFA production across autoimmune diseases.

Disease	SCFA Levels (Butyrate, Acetate, Propionate)	SCFA-Producing Bacteria (Faecalibacterium, Roseburia, Bifidobacterium, Lactobacillus)	Pro-Inflammatory Bacteria (Proteobacteria, Escherichia-Shigella, Bacteroides)	Tight Junction Proteins (Claudins & Occludins)	Zonulin (Gut Permeability Marker)
**Type 1 Diabetes (T1D) (** 34, 43)	↓ Reduced	↓ Reduced	↑ Increased	↓ Reduced	↑ Elevated
**Celiac Disease (** 44, 47–49)	↓ Reduced	↓ Reduced	↑ Increased	↓ Reduced	↑ Elevated
**Hashimoto’s Thyroiditis (** 50, 51)	↓ Reduced	↓ Reduced	↑ Increased	↓ Reduced	↑ Elevated
**Rheumatoid Arthritis (RA**) (52–54)	↓ Reduced	↓ Reduced	↑ Increased	↓ Reduced	↑ Elevated
**Multiple Sclerosis (MS) (** 52)	↓ Reduced	↓ Reduced	↑ Increased	↓ Reduced	↑ Elevated
**Systemic Lupus Erythematosus (SLE) (** 55)	↓ Reduced	↓ Reduced	↑ Increased	↓ Reduced	↑ Elevated
**Addison’s Disease (** 13)	↓ Reduced	↓ Reduced	↑ Increased	↓ Reduced	↑ Elevated

This table summarizes the impact of gut microbiota composition, SCFA metabolism, and intestinal barrier integrity in autoimmune diseases. SCFA levels (butyrate, acetate, propionate) are consistently reduced across all listed diseases, reflecting a decrease in beneficial bacteria such as Faecalibacterium, Roseburia, Bifidobacterium, and Lactobacillus. In contrast, pro-inflammatory bacterial taxa (Proteobacteria, Escherichia-Shigella, Bacteroides) are elevated, which may contribute to chronic inflammation and immune dysregulation.

Tight junction proteins (claudins & occludins) are significantly reduced, indicating increased intestinal permeability (“leaky gut”), while zonulin levels are elevated, further supporting the presence of compromised gut barrier function. These findings are particularly pronounced in T1D, celiac disease, and Hashimoto’s thyroiditis, where barrier dysfunction plays a key role in disease pathogenesis. Similar patterns are observed in RA, MS, SLE, and Addison’s disease, underscoring gut permeability as a common factor in autoimmunity.

These data are derived from a combination of human clinical studies, animal models, and *in vitro* research. Further large-scale studies integrating microbiota profiling with functional markers of gut permeability and inflammation are necessary to better understand the systemic effects of gut dysbiosis in autoimmune diseases.

Future research must emphasize longitudinal studies and multi-omics analyses designed to elucidate precise molecular mechanisms linking specific environmental exposures, microbiota changes, and ECS dysfunction (3, 10). Comprehensive characterization of microbial metabolite profiles, ECS receptor expression patterns, and systemic inflammatory markers in at-risk populations could yield predictive biomarkers identifying individuals progressing toward autoimmune disease (33, 35). Furthermore, developing targeted therapeutic strategies aimed explicitly at preventing ECS receptor desensitization (6) or reversing dysbiosis-induced ECS dysfunction (4, 12) holds substantial promise for autoimmune disease prevention and management.

In conclusion, environmental factors play an indispensable role in manifesting latent genetic susceptibility to T1D through mechanisms centered around gut dysbiosis and ECS dysfunction (3, 13). The ECBoM framework provides an integrative perspective illustrating how pervasive environmental influences, commonplace in modern lifestyles, systematically degrade gut microbiome resilience and ECS homeostasis, facilitating autoimmune disease initiation and progression (4, 19). Effective interventions targeting gut microbiota restoration and ECS stabilization represent critical opportunities for reducing autoimmune risk, particularly in genetically susceptible individuals, marking a promising frontier in autoimmune disease prevention and management (28, 30).

## ECS dysfunction across key tissues in T1D pathogenesis

The endocannabinoid system (ECS) functions as a critical regulator of physiological homeostasis across multiple tissues implicated in type 1 diabetes (T1D) pathogenesis, including pancreatic β-cells, intestinal epithelium, vascular endothelium, and immune tissues (1, 25). Chronic ECS dysregulation, induced primarily by persistent environmental triggers and gut dysbiosis, profoundly compromises the physiological functions of these tissues, promoting autoimmune activation and accelerating disease progression (3, 8). Understanding tissue-specific ECS dysfunction mechanisms is fundamental to fully appreciating the integrative pathology proposed by the ECBoM hypothesis. **Table 2** summarizes ECS-related receptor expression patterns and calcium disturbances across tissues affected in autoimmune diseases.

**Table 2:** ECS-related disturbances across autoimmune diseases.

Disease	Tissue of Analysis	AEA	2-AG	FAAH (Fatty Acid Amide Hydrolase)	MAGL (Monoacylglycerol Lipase)	CB1 Expression	CB2 Expression	TRPV1 Expression	TRPV2 Expression	Calcium Level (Blood)
**Type 1 Diabetes (T1D) (** 15, 16)	Plasma, pancreas, immune cells	↓ Lowered	↑ Elevated	↓ Lowered (Pancreas, Gut)	↑ Increased (Pancreas, Gut)	↑ Increased (Brain, Pancreas)	↓ Lowered (Pancreas, Immune Cells)	↓ Lowered (Pancreas, Gut, Thyroid)	↓ Lowered (Pancreas, Gut, Thyroid)	↓ Lowered
**Hashimoto’s Thyroiditis (** 51)	Thyroid, plasma	↓ Lowered	↑ Elevated	↓ Lowered (Thyroid)	↑ Increased (Thyroid)	↑ Increased (Thyroid)	↑ Increased (Thyroid)	↓ Lowered (Thyroid)	↓ Lowered (Thyroid)	↓ Lowered
**Celiac Disease** (29)	Small intestine, plasma	↓ Lowered	↑ Elevated	↓ Lowered (Gut Mucosa)	↑ Increased (Gut Mucosa)	↑ Increased (Gut Mucosa)	↑ Increased (Gut Mucosa)	↓ Lowered (Gut Mucosa)	↓ Lowered (Gut Mucosa)	↓ Lowered
**Rheumatoid Arthritis (RA) (** 56)	Synovial tissue, plasma	↓ Lowered	↑ Elevated	↓ Lowered (Synovium)	↑ Increased (Synovium)	↑ Increased (Synovium)	↑ Increased (Synovium)	↓ Lowered (Joints, Synovium)	↓ Lowered (Joints, Synovium)	↓ Lowered
**Multiple Sclerosis (MS) (** 57)	Brain, spinal cord, plasma	↑ Elevated	↑ Elevated	No Change (Brain, Spinal Cord)	No Change (Brain, Spinal Cord)	↑ Increased (Brain, Spinal Cord)	↑ Increased (Brain, Spinal Cord)	↓ Lowered (Brain, Spinal Cord)	↓ Lowered (Brain, Spinal Cord)	↓ Lowered
**Systemic Lupus Erythematosus (SLE) (** 36)	Plasma, immune cells	No Change	↑ Elevated	↑ Increased (Kidney, Skin)	No Change (Kidney, Skin)	↑ Increased (Kidney, Skin)	↑ Increased (Kidney, Skin)	↓ Lowered (Kidney, Skin)	↓ Lowered (Kidney, Skin)	↓ Lowered
**Addison’s Disease (** 13, 16)	Adrenal gland, plasma	↓ Lowered	↓ Lowered	↓ Lowered (Adrenal Gland)	↓ Lowered (Adrenal Gland)	↑ Increased (Adrenal Gland)	↓ Lowered (Adrenal Gland)	↓ Lowered (Adrenal Gland)	↓ Lowered (Adrenal Gland)	↓ Lowered

This table summarizes ECS-related biomarker alterations in autoimmune diseases, based on findings from human studies, animal models, and *in vitro* experiments. Across conditions, disruptions in AEA, 2-AG, and receptor expression suggest a widespread role for ECS in immune regulation and metabolic homeostasis.

In type 1 diabetes (T1D), elevated 2-AG and reduced AEA levels coincide with increased CB1 expression in the brain and pancreas, along with decreased CB2 in immune and pancreatic tissues, potentially contributing to β-cell dysfunction. Similar trends in ECS dysregulation appear in celiac disease and Hashimoto’s thyroiditis, where altered endocannabinoid levels and receptor expression may influence gut permeability and thyroid autoimmunity.

Rheumatoid arthritis (RA) and multiple sclerosis (MS) exhibit ECS disturbances in inflamed joints and the central nervous system, respectively, with increased CB1 expression and reduced TRPV1/2, pointing to a role in chronic inflammation. In systemic lupus erythematosus (SLE), elevated 2-AG and altered CB1/CB2 expression suggest a complex interplay between ECS and systemic immune dysregulation. Addison’s disease, primarily affecting adrenal function, shows reduced endocannabinoid levels and CB2 expression, implicating ECS in steroidogenesis and immune modulation.

Disruptions in calcium homeostasis across these conditions indicate a potential link between ECS dysfunction and broader metabolic disturbances. While animal models provide valuable insights, further human studies are needed to clarify ECS’s role in autoimmunity and its potential as a therapeutic target.

Pancreatic β-cells are central to T1D pathogenesis due to their unique susceptibility to autoimmune destruction. These insulin-producing cells express functional ECS receptors, notably CB1, CB2, and prominently TRPV1 channels, that modulate cellular metabolism, insulin secretion, inflammation, and survival signaling (1, 15). Under normal physiological conditions, ECS signaling, especially via CB2 and TRPV1, provides essential regulatory input that maintains β-cell viability, mitigates inflammatory stress, and stabilizes mitochondrial functions (32). However, chronic elevation of 2-arachidonoylglycerol (2-AG), resulting from dysbiosis-induced ECS hyperactivation, profoundly disrupts these protective regulatory pathways (3). Persistent TRPV1 overstimulation rapidly induces receptor desensitization, impairing critical calcium influx necessary for insulin secretion and mitochondrial integrity (17, 24). Dysfunctional calcium signaling destabilizes mitochondrial membranes, induces reactive oxygen species (ROS) generation, and promotes endoplasmic reticulum (ER) stress, activating apoptotic pathways within β-cells. Concomitantly, reduced CB2 receptor responsiveness due to receptor internalization exacerbates inflammation and diminishes anti-inflammatory cytokine release (notably IL-10), rendering β-cells vulnerable to autoimmune-mediated destruction (16, 25). Thus, chronic ECS dysregulation directly amplifies β-cell stress, dysfunction, and susceptibility to autoimmunity, critically accelerating T1D progression.

ECS dysfunction similarly affects intestinal epithelial cells, essential regulators of gut permeability, immune tolerance, and systemic inflammation. Intestinal epithelia abundantly express CB2 and TRPV1 receptors, whose activation tightly controls intestinal barrier integrity, epithelial regeneration, mucus secretion, and antimicrobial peptide production (8, 17). Physiologically, ECS signaling strengthens epithelial tight junction proteins, thereby preserving barrier function and limiting systemic exposure to luminal inflammatory stimuli (3). Dysbiotic microbiota perturbations, however, elevate intestinal 2-AG levels, inducing chronic ECS receptor desensitization, predominantly TRPV1 (3, 25). Impaired TRPV1 signaling disrupts calcium homeostasis, directly compromising epithelial tight junction stability and reducing mucus barrier functionality, significantly enhancing intestinal permeability (17). Elevated intestinal permeability (leaky gut) facilitates translocation of microbial components such as lipopolysaccharides (LPS) and dietary antigens into systemic circulation, triggering systemic inflammation and immune dysregulation (11, 13). Additionally, ECS dysfunction reduces epithelial regenerative capacity by limiting proliferation signaling, impairing intestinal resilience, and exacerbating mucosal inflammation (3, 22). These barrier disruptions perpetuate systemic inflammation, directly linking gut ECS dysfunction to autoimmune pathogenesis.

The vascular endothelium represents another critical tissue profoundly impacted by ECS dysfunction. Endothelial cells express abundant TRPV1 and cannabinoid receptors (especially CB2), which regulate endothelial barrier permeability, leukocyte extravasation, inflammatory signaling, and vascular homeostasis (3, 23). Under physiological conditions, ECS signaling via CB2 and TRPV1 maintains endothelial integrity by stabilizing intercellular junctions, limiting inflammatory mediator expression, and restricting leukocyte trafficking (23). Persistent ECS overstimulation induced by chronic microbial dysbiosis significantly impairs endothelial receptor signaling, notably via TRPV1 receptor desensitization (17, 25). This receptor-level dysfunction compromises calcium-dependent endothelial barrier functions, increasing endothelial permeability and facilitating immune cell infiltration into pancreatic islets and intestinal tissues (23). Dysfunctional endothelial cells further amplify local inflammatory responses by secreting chemokines (e.g., MCP-1) and adhesion molecules (ICAM-1, VCAM-1), actively recruiting autoreactive T-cells and macrophages into target tissues (23). Consequently, vascular ECS dysfunction critically exacerbates autoimmune inflammatory infiltration, significantly accelerating β-cell destruction and overall disease progression.

Immune tissues and cells are similarly vulnerable to chronic ECS dysfunction, profoundly affecting immune regulation and autoimmune tolerance. Regulatory T-cells (Tregs), essential suppressors of autoimmunity, depend heavily on ECS signaling, especially CB2 activation, for their differentiation, stability, and functional integrity (8, 25). Physiological ECS stimulation promotes Treg induction through enhanced FOXP3 expression, stabilizes immunosuppressive cytokine profiles (IL-10, TGF-β), and restricts inflammatory Th17 cell differentiation (25, 29). However, persistent ECS receptor overstimulation caused by chronic microbiota-driven ECS hyperactivity induces receptor desensitization (notably CB2), diminishing Treg differentiation, stability, and function (16). The resultant depletion of functional Tregs directly compromises immune tolerance, enabling uncontrolled autoreactive T-cell activation and pro-inflammatory cytokine release (IL-17, IL-23), critically fueling autoimmune processes (16). Additionally, ECS dysfunction alters macrophage polarization toward pro-inflammatory (M1) phenotypes, exacerbating systemic inflammatory responses and further amplifying tissue-specific autoimmune damage (8, 16, 29).

Importantly, ECS dysfunction across these tissues does not occur in isolation; instead, it represents an interconnected pathogenic cascade driven by chronic environmental and microbial perturbations. For example, dysbiosis-induced gut ECS dysfunction increases systemic inflammation and intestinal permeability (3, 4, 12), exacerbating endothelial ECS impairment (23, 42) and promoting immune infiltration into pancreatic islets (1, 15, 20). Concurrently, β-cell ECS dysregulation amplifies local inflammatory responses (16, 58), increasing antigen exposure and immune activation (8, 25). This self-sustaining, tissue-spanning inflammatory cascade underscores ECS dysfunction as a central integrative pathology within T1D.

Therapeutically targeting tissue-specific ECS dysfunction thus offers compelling potential for interrupting disease progression. Pharmacological interventions designed to prevent ECS receptor desensitization (e.g., selective TRPV1 modulators or CB2 agonists) could restore ECS signaling, preserving β-cell viability (15, 58), intestinal barrier integrity (3, 42), endothelial function (23), and immune tolerance (8, 16). Additionally, strategies targeting endocannabinoid metabolism enzymes (DAGL, MAGL) to regulate systemic 2-AG levels (14, 22) may prevent chronic ECS overstimulation, thereby interrupting pathogenic inflammatory cascades (1, 4). Given ECS’s integrative regulatory capacity, these targeted therapeutic interventions hold significant promise for mitigating tissue-specific autoimmune damage, preserving immune tolerance, and ultimately preventing or delaying clinical diabetes manifestation.

In conclusion, ECS dysfunction across pancreatic β-cells, intestinal epithelial cells, vascular endothelial cells, and immune tissues constitutes a critical, integrative molecular pathology underpinning T1D progression within the ECBoM hypothesis framework (1, 3). Chronic ECS receptor desensitization—driven by microbiota dysbiosis-induced ECS overstimulation—critically impairs tissue-specific functions (4, 9), exacerbating inflammatory, metabolic, and autoimmune pathologies (8, 42, 58). Elucidating tissue-specific ECS dysregulation mechanisms provides compelling insights into autoimmune diabetes pathogenesis and identifies novel therapeutic targets aimed at preserving ECS functionality and preventing T1D progression (15, 16).

## Molecular pathology of ECS dysfunction: receptor desensitization and TRPV1 susceptibility

The prolonged elevation of endocannabinoid levels, particularly 2-arachidonoylglycerol (2-AG), contributes significantly to the pathogenesis of type 1 diabetes (T1D) through mechanisms involving desensitization of key receptors within the endocannabinoid system (ECS) (22, 23). ECS receptors, including cannabinoid receptor type 1 (CB1), cannabinoid receptor type 2 (CB2), and the transient receptor potential vanilloid type-1 (TRPV1) channel, orchestrate complex cellular signaling pathways essential for immune regulation, inflammatory control, and metabolic homeostasis (5, 6). Under physiological conditions, ECS receptor activation is transient, followed rapidly by mechanisms such as receptor phosphorylation, internalization, and recycling, processes designed to prevent overstimulation and cellular dysfunction (59). However, continuous receptor stimulation through persistently elevated 2-AG levels (7) induces chronic receptor desensitization (7, 9, 60). This maladaptive phenomenon involves receptor phosphorylation mediated by intracellular kinases, notably G protein-coupled receptor kinases (GRKs), protein kinase C (PKC), and protein kinase A (PKA), followed by β-arrestin binding, internalization, and eventual receptor degradation or recycling impairment (16). As a result, chronic receptor desensitization substantially reduces ECS signaling efficacy, compromising the critical immunoregulatory and anti-inflammatory actions exerted by these receptors (8). Although CB1, CB2, and TRPV1 all respond to elevated 2-AG levels, experimental studies consistently demonstrate that TRPV1 is the most susceptible to rapid desensitization, followed by CB1 and CB2 in descending order of sensitivity (15–17).

Significantly, ECS receptors exhibit marked differences in their vulnerability to prolonged agonist exposure. Among these, the TRPV1 receptor displays the highest susceptibility and the fastest kinetics of desensitization when persistently activated by elevated 2-AG (17). TRPV1, unlike CB1 and CB2, is a calcium-permeable cation channel rather than a classical G protein-coupled receptor (15). Upon activation, TRPV1 channels induce immediate calcium influx, triggering intracellular kinase cascades and subsequent receptor phosphorylation, rapidly reducing channel responsiveness (16). Experimental evidence consistently demonstrates that prolonged exposure to elevated 2-AG concentrations causes a swift decline in TRPV1 activity, profoundly disrupting calcium homeostasis and subsequent intracellular signaling pathways (15, 17).

The distinct sensitivity of TRPV1 to desensitization becomes critically significant due to the receptor’s unique expression profile. TRPV1 channels are abundantly expressed in several tissues central to the ECBoM hypothesis of T1D pathogenesis, such as pancreatic β-cells, intestinal epithelial cells, vascular endothelial cells, and immune cell subsets (15, 17). Pancreatic β-cells, in particular, depend extensively on finely tuned calcium signaling for their metabolic function, insulin secretion, mitochondrial integrity, and cellular survival (24). Chronic TRPV1 desensitization in these cells disrupts essential calcium signaling, precipitating mitochondrial dysfunction, endoplasmic reticulum stress, and enhanced apoptotic susceptibility (16). This cellular vulnerability amplifies β-cell susceptibility to inflammatory cytokines and autoimmune attack, accelerating autoimmune destruction and progression toward overt T1D (15).

Furthermore, intestinal epithelial cells, another tissue type prominently expressing TRPV1, rely heavily on calcium-mediated signaling for barrier maintenance and immunoregulatory functions (17). Persistent desensitization of TRPV1 channels in intestinal epithelia impairs calcium-dependent tight junction stability, compromising intestinal barrier integrity (3). Such impairment facilitates increased intestinal permeability, allowing the translocation of bacterial antigens, lipopolysaccharides, and inflammatory mediators into systemic circulation, further exacerbating systemic inflammation and immune dysregulation (11, 17, 19). Thus, the rapid desensitization of TRPV1 in the gut represents a critical nexus linking gut dysbiosis-induced ECS dysfunction with systemic autoimmune and inflammatory responses central to T1D development.

In addition to TRPV1-mediated disruptions, accumulating evidence highlights the pivotal role of mitochondrial cannabinoid receptors (mtCB1 and mtCB2) in mediating intracellular stress responses and metabolic reprogramming in target cells (6, 22). Endocannabinoids, particularly 2-AG, readily diffuse across cellular and organelle membranes due to their lipophilic nature, accumulating in mitochondrial membranes (7). mtCB1 receptors, expressed predominantly on the outer mitochondrial membrane (OMM) and potentially also on the inner membrane (IMM), modulate mitochondrial respiration, membrane potential (ΔΨm), and reactive oxygen species (ROS) generation (22). Persistent stimulation of mtCB1 by elevated 2-AG impairs mitochondrial oxidative phosphorylation, reduces ATP production, and promotes mitochondrial dysfunction—a pathological cascade especially detrimental to pancreatic β-cells and immune cells reliant on oxidative metabolism (15, 58). In β-cells, mtCB1 overstimulation compromises mitochondrial integrity and calcium handling, synergizing with TRPV1 desensitization to intensify apoptotic vulnerability and impair insulin secretion (58). In immune cells, ECS-mediated mitochondrial reprogramming shifts cellular metabolism toward glycolysis, inhibiting Treg stability and favoring pro-inflammatory phenotypes (e.g., Th17 and M1 macrophages), thereby exacerbating immune imbalance (8, 25). Similarly, mtCB2 receptors may influence mitochondrial survival pathways and ROS buffering in macrophages and dendritic cells, with dysfunctional mtCB2 signaling contributing to impaired M2 polarization and excessive pro-inflammatory activation (25). These findings underscore the role of ECS-driven mitochondrial dysfunction as a central mechanism coupling chronic endocannabinoid elevation with immune and metabolic dysregulation in T1D pathogenesis.

Similar mechanisms pertain to vascular endothelial cells, which prominently express TRPV1 channels and utilize calcium influx to regulate vascular permeability, endothelial cell survival, and leukocyte trafficking (23, 61). Chronic TRPV1 desensitization within endothelial cells compromises endothelial integrity, enhancing leukocyte extravasation into target tissues, notably pancreatic islets. Such augmented immune cell infiltration substantially increases local inflammation, promoting autoimmune activation and pancreatic β-cell destruction, thus actively contributing to the autoimmune pathogenesis of T1D (23, 62).

In the face of chronic TRPV1 desensitization, the ECS attempts to maintain physiological homeostasis through compensatory adjustments involving cannabinoid receptors CB1 and CB2 (25, 40). Initially, CB2 receptors exert significant anti-inflammatory and immunosuppressive effects, supporting regulatory T-cell stability and limiting pro-inflammatory cytokine release (8, 25). However, persistent ECS overstimulation eventually leads to compensatory CB2 receptor desensitization and functional impairment, diminishing this anti-inflammatory response (56). Concurrently, CB1 receptor activity becomes dysregulated, amplifying metabolic dysfunction and inflammatory signaling (22). Ultimately, this progressive receptor dysfunction erodes ECS buffering capacity, tipping immunological equilibrium from regulated tolerance toward autoimmunity and overt inflammation (46).

Recognizing the central role of receptor desensitization—especially the particular vulnerability of TRPV1—offers compelling therapeutic implications. Therapeutic strategies aimed at stabilizing TRPV1 receptor responsiveness without provoking chronic desensitization could prevent the downstream cellular and physiological consequences described above (16, 17). For instance, selective TRPV1 modulators, including partial agonists or antagonists designed to stabilize receptor function, may represent novel therapeutic interventions (17). Additionally, targeted modulation of endocannabinoid metabolism—specifically, inhibiting enzymes such as diacylglycerol lipase (DAGL) or monoacylglycerol lipase (MAGL)—to reduce chronic 2-AG elevation could represent another promising strategy to prevent receptor overstimulation and subsequent desensitization (14, 46).

Future research should prioritize detailed elucidation of TRPV1 desensitization dynamics, intracellular molecular cascades, receptor recycling pathways, and strategies to pharmacologically modulate receptor activity effectively (14, 17). Longitudinal studies employing advanced multi-omics approaches to profile ECS receptor expression and function in genetically predisposed or high-risk populations may identify early markers predictive of T1D progression (3, 36). Integration of these findings into clinical practice could facilitate targeted early interventions designed to stabilize ECS signaling, delay or prevent autoimmune progression, and ultimately improve clinical outcomes in genetically susceptible individuals (7, 25).

In conclusion, ECS receptor desensitization—highlighted by the unique sensitivity of TRPV1 to chronic 2-AG overstimulation—represents a pivotal molecular pathology within the ECBoM model of T1D pathogenesis (14, 17). Addressing this receptor-specific desensitization therapeutically could provide powerful strategies for restoring ECS homeostasis, preserving immune-metabolic regulation, and ultimately preventing or delaying the clinical manifestation of autoimmune diabetes (3, 7, 25).

## Immunological escalation & loss of tolerance

Autoimmune diabetes emerges through a progressive escalation of immune dysregulation characterized by compromised peripheral tolerance, aberrant immune cell activation, and chronic inflammatory cascades targeting pancreatic β-cells (63, 64). Central to this pathological progression is the chronic dysregulation of the endocannabinoid system (ECS), critically modulated by gut microbiota-derived short-chain fatty acids (SCFAs), particularly butyrate, and endocannabinoids, notably 2-arachidonoylglycerol (2-AG) (3, 65). The dysbiotic gut environment, featuring marked reductions in beneficial butyrate-producing bacterial species, results in diminished SCFA availability and consequent intestinal ECS dysregulation, laying the foundation for sustained systemic inflammation and profound immunological disturbances integral to autoimmune escalation and loss of immune tolerance (66).

Under physiological conditions, SCFAs—particularly butyrate—derived from beneficial gut microbial metabolism exert potent immunomodulatory effects within intestinal mucosa, enhancing regulatory T-cell (Treg) differentiation and stability, promoting anti-inflammatory cytokine production (IL-10, TGF-β), and supporting epithelial barrier integrity (67, 68). These effects are critically mediated through activation of peroxisome proliferator-activated receptors (PPARs), primarily PPARγ, expressed in immune cells, intestinal epithelia, and vascular endothelial cells (69, 70). PPARγ signaling enhances Treg induction via transcriptional upregulation of FOXP3, mitigates inflammatory cytokine expression, and fortifies epithelial tight junctions (70). Butyrate and other SCFAs thus represent pivotal microbiota-derived mediators linking microbiota composition directly to systemic immune homeostasis (70).

Chronic microbiota dysbiosis, however, characterized by substantial depletion of butyrate-producing bacteria (such as *Faecalibacterium prausnitzii*, *Roseburia intestinalis*) drastically diminishes SCFA availability, critically reducing PPARγ signaling, Treg differentiation, and epithelial barrier integrity (71, 72). Reduced SCFA levels directly exacerbate intestinal permeability, increasing the systemic translocation of luminal antigens and microbial endotoxins, particularly lipopolysaccharide (LPS), thus significantly amplifying inflammatory signaling via toll-like receptors (TLR4) and nuclear factor-kappa B (NF-κB) pathways (70, 73). Concurrently, decreased PPARγ signaling impairs immune regulatory networks, facilitating a shift from protective Treg-dominated responses toward pathogenic Th17 cell responses, intensifying systemic inflammation and autoimmunity (70).

Critically, reduced SCFA levels and heightened systemic LPS influx result in ECS dysregulation, primarily through profound alterations in intestinal and systemic endocannabinoid profiles, notably elevated 2-AG levels (12, 74). Elevated LPS robustly induces diacylglycerol lipase (DAGL) activity, enhancing 2-AG synthesis, contributing to persistent ECS receptor overstimulation (particularly TRPV1 and CB1) (14, 75). As previously discussed, chronic ECS receptor overstimulation leads to receptor desensitization and impaired downstream signaling in intestinal epithelial cells and immune cells, exacerbating gut barrier dysfunction, immune cell activation, and pro-inflammatory cytokine production (76).

Moreover, chronic inflammation within immune compartments further amplifies 2-AG overproduction. Monocytes, macrophages, and dendritic cells exposed to persistent inflammatory stimuli—particularly LPS—upregulate DAGL-β, the enzyme primarily responsible for peripheral 2-AG synthesis (7, 8). This leads to sustained paracrine and autocrine 2-AG signaling in immune microenvironments. Although CB2 activation by 2-AG typically exerts anti-inflammatory effects (25, 40), chronic overexposure may paradoxically impair CB2 receptor responsiveness, disrupt Treg differentiation, and favor Th1/Th17 skewing (8, 29), thus undermining immune regulation and accelerating autoimmunity. This feedback loop of inflammatory ECS amplification represents a crucial, yet underrecognized, axis of immune escalation and tolerance breakdown in T1D (1, 16).

The resultant ECS dysfunction significantly impairs local intestinal immune homeostasis, particularly compromising Treg induction, stability, and suppressive functionality (8, 25). Reduced CB2 receptor responsiveness critically diminishes Treg differentiation by limiting FOXP3 transcriptional activation and anti-inflammatory cytokine secretion, impairing peripheral tolerance (29, 40). Furthermore, ECS dysfunction disrupts macrophage polarization, skewing macrophage profiles toward pro-inflammatory (M1) phenotypes characterized by elevated secretion of TNF-α, IL-1β, and IL-6, thereby intensifying local and systemic inflammatory responses (1, 46). Simultaneously, impaired ECS signaling shifts dendritic cells (DCs) toward immunostimulatory phenotypes, enhancing their antigen-presenting capacity and promoting autoreactive T-cell activation, particularly pathogenic Th17 cells producing IL-17 and IL-23 (25, 56). This pro-inflammatory shift critically weakens mucosal tolerance mechanisms, facilitating systemic autoimmunity (8, 22).

Concurrently, ECS dysregulation profoundly impacts vascular endothelial cells in intestinal mucosa, impairing endothelial barrier functions and exacerbating leukocyte trafficking into peripheral tissues (1, 23). Endothelial dysfunction mediated through TRPV1 desensitization critically amplifies leukocyte extravasation by upregulating endothelial adhesion molecules (ICAM-1, VCAM-1) and inflammatory chemokines (e.g., MCP-1), thus actively recruiting autoreactive lymphocytes and macrophages to pancreatic islets and intestinal tissues (17, 62). Enhanced leukocyte infiltration triggers local inflammatory cascades characterized by cytokine production (IFN-γ, IL-17, TNF-α), cytotoxic T-cell activation, and tissue-specific autoimmune responses targeting insulin-producing β-cells (1, 8).

Ultimately, sustained inflammatory cascades driven by impaired ECS and diminished SCFA-PPARγ signaling culminate in the progressive loss of immune tolerance (3, 10). Initial tolerance breakdown manifests through diminished Treg activity, followed by escalated autoreactive T-cell activation and clonal expansion (8, 25). Chronic inflammatory stimuli further amplify autoreactive lymphocyte populations, intensifying autoimmune responses and irreversibly damaging pancreatic β-cells (1, 31). This immunological escalation rapidly surpasses ECS’s remaining compensatory capacities, firmly transitioning from latent autoimmune processes to overt clinical autoimmune diabetes (4, 43).

Therapeutically, targeting critical nodes within this immunological escalation pathway—such as enhancing SCFA availability, augmenting PPARγ activation, or pharmacologically restoring ECS receptor responsiveness—represents promising strategies to restore immune homeostasis and peripheral tolerance (3, 10). Dietary and probiotic interventions specifically designed to replenish SCFA-producing microbiota populations could significantly bolster mucosal Treg induction and reinforce epithelial barrier integrity (27, 28). Similarly, pharmacological agents directly activating PPARγ signaling, or selective ECS modulators aimed at stabilizing receptor responsiveness (e.g., CB2 agonists, TRPV1 partial modulators, inhibitors of 2-AG synthesis enzymes), could effectively restore local ECS signaling, reducing inflammation, preserving mucosal immune homeostasis, and preventing autoimmune progression (1, 25, 40).

Future studies should emphasize detailed mechanistic characterization of SCFA-PPARγ-ECS interactions in genetically predisposed or at-risk populations, employing advanced multi-omics approaches (3, 10). Longitudinal profiling of SCFA levels, ECS receptor activity, immune cell differentiation, and cytokine responses may identify predictive biomarkers capable of distinguishing individuals progressing toward overt autoimmune diabetes (25, 33). Integration of these predictive biomarkers into clinical practice could enable early, targeted preventive interventions aimed explicitly at restoring ECS and immune homeostasis, ultimately improving clinical outcomes in genetically susceptible populations (4, 28).

In conclusion, immunological escalation and loss of peripheral tolerance in T1D fundamentally involve chronic microbiota-driven depletion of beneficial SCFA-producing bacterial populations, reduced PPARγ signaling, subsequent ECS dysregulation, and sustained inflammatory cascades (3, 8, 10). Addressing SCFA-ECS-immune interactions thus represents a compelling therapeutic approach for restoring immunological balance, preventing autoimmune escalation, and delaying or even reversing the progression of clinical autoimmune diabetes (1, 4, 25).

## Parallels to other autoimmune disorders

Type 1 diabetes (T1D) frequently coexists with a well-established cluster of autoimmune conditions, notably celiac disease (CD), Hashimoto’s thyroiditis (HT), Addison’s disease (AAD), and rheumatoid arthritis (RA). While each disease presents unique clinical manifestations and targets distinct tissues, accumulating evidence underscores shared molecular disturbances involving the endocannabinoid system (ECS), particularly alterations in transient receptor potential vanilloid channels (TRPV1 and TRPV2), cannabinoid receptor type 2 (CB2), and underlying genetic susceptibility (29, 42, 56). Identifying common ECS-mediated mechanisms and genetic predispositions elucidates critical integrative pathways that potentially drive autoimmune processes across these conditions, reinforcing the plausibility of the ECBoM hypothesis in T1D pathogenesis (25, 40).

Among these shared molecular mechanisms, the prominence of TRPV channels, particularly TRPV1 and TRPV2, is striking. TRPV1 and TRPV2 are calcium-permeable, nonselective cation channels abundantly expressed in endocrine and exocrine tissues, where regulated calcium influx is essential for proper cellular function, survival, and secretion (17, 24). Crucially, sustained elevations in endocannabinoid levels, notably 2-arachidonoylglycerol (2-AG), common in dysbiotic and inflammatory states, render these channels vulnerable to rapid receptor desensitization (22, 46). Chronic TRPV1/2 desensitization disrupts calcium homeostasis, mitochondrial integrity, endoplasmic reticulum function, and cellular survival pathways, thereby critically compromising the viability of hormone-producing and barrier-regulating cells within affected tissues (29, 42).

Celiac disease, frequently co-occurring with T1D, exemplifies this phenomenon. CD is characterized by autoimmune-mediated intestinal epithelial damage driven by gluten exposure, substantially impairing gut barrier function (47, 48). Significantly, intestinal epithelial cells in CD exhibit high TRPV1 expression, rendering them particularly sensitive to chronic ECS overstimulation and rapid receptor desensitization upon elevated 2-AG exposure (42, 46). TRPV1 dysfunction within intestinal epithelial cells impairs calcium-dependent regulation of tight junction proteins, mucus secretion, and epithelial regenerative capacity, exacerbating intestinal permeability and systemic inflammation (9, 29). Concurrently, reduced CB2 receptor expression in CD further compounds immunological dysregulation, limiting anti-inflammatory and regulatory T-cell induction crucial for mucosal tolerance (42, 56). These ECS disturbances align precisely with mechanisms proposed for T1D, emphasizing shared ECS-mediated vulnerabilities between CD and T1D (49).

Hashimoto’s thyroiditis, another common autoimmune disorder co-occurring with T1D, further highlights shared ECS dysfunction involving TRPV1/2 and CB2 receptors. The thyroid gland exhibits abundant expression of TRPV1 and TRPV2 channels, critical for calcium-regulated thyroid hormone synthesis, secretion, and epithelial cell survival (17, 61). Chronic inflammation observed in HT corresponds with significantly elevated 2-AG levels and subsequent TRPV1 receptor overstimulation, leading to rapid receptor desensitization, disrupted calcium homeostasis, mitochondrial dysfunction, and thyroid follicular cell apoptosis (1, 17). Genetic analyses in HT populations have identified polymorphisms in the TRPV1 gene (e.g., rs8065080), potentially linking impaired receptor function to increased thyroid autoimmunity risk (17, 50). Concurrent CB2 receptor dysregulation reduces anti-inflammatory signaling within thyroid tissue, perpetuating inflammatory processes (25, 56). Thus, thyroid ECS dysregulation parallels mechanisms seen in pancreatic β-cells in T1D, supporting a shared pathogenic ECS model.

Similarly, autoimmune Addison’s disease, characterized by autoimmune-mediated destruction of adrenal cortical tissue, provides further compelling evidence of shared ECS-related autoimmune vulnerabilities. Adrenal cortical cells critically rely on TRPV1 and TRPV2 channels for regulated calcium influx, essential for steroidogenesis and mitochondrial energy production (17, 61). Chronic systemic inflammation and elevated endocannabinoid levels in AAD patients contribute significantly to rapid TRPV receptor desensitization and impaired calcium signaling (1, 46). Dysfunctional calcium homeostasis within adrenal cortical cells triggers mitochondrial dysfunction, ROS accumulation, and apoptosis, accelerating autoimmune-mediated adrenal damage. Genetic susceptibility studies in autoimmune adrenalitis patients have also highlighted polymorphisms in ECS-related genes, including CNR2 and TRPV1, suggesting shared ECS-driven genetic predispositions among these autoimmune disorders, further aligning with T1D susceptibility profiles (25, 50, 56).

Rheumatoid arthritis, although less commonly associated with T1D, demonstrates significant ECS dysregulation, particularly involving TRPV1/2 and CB2 receptors within synovial tissues. Synovial fibroblasts and inflammatory infiltrating immune cells abundantly express TRPV channels, whose chronic desensitization due to elevated 2-AG significantly disrupts calcium signaling, cellular proliferation, apoptosis regulation, and inflammatory mediator production (17, 22). Synovial inflammation and joint destruction in RA directly correlate with ECS dysregulation and diminished CB2 receptor responsiveness, exacerbating inflammatory cytokine release (IL-17, IL-6, TNF-α), leukocyte recruitment, and chronic tissue damage (16, 56). Genetic predisposition studies in RA have similarly identified variants in ECS-related genes (e.g., FAAH, TRPV1), further supporting the integrative role of ECS genetics in autoimmune vulnerability (12, 17).

These examples clearly demonstrate ECS dysregulation, particularly chronic TRPV1/2 receptor desensitization, calcium signaling disruption, and impaired CB2-mediated immune regulation as common pathological features among T1D and coexisting autoimmune disorders (16, 17, 56). Additionally, the consistent observation of gut microbiota dysbiosis—characterized by reduced SCFA/butyrate-producing populations, increased intestinal permeability, and elevated systemic inflammation—further reinforces ECS dysfunction across these diseases (10, 27, 43). Reduced intestinal SCFA availability critically diminishes peroxisome proliferator-activated receptor gamma (PPARγ)-mediated immunoregulatory pathways, enhancing ECS dysfunction and systemic autoimmune responses (3, 8, 12).

From a genetic perspective, autoimmune polyendocrine syndrome type 2 (APS-2), characterized by coexistence of T1D, AAD, and HT, strongly implicates shared genetic susceptibility loci involving ECS-related genes. Genome-wide association studies (GWAS) have identified overlapping susceptibility regions in these conditions, notably within HLA regions but increasingly involving non-HLA genes linked to immune regulation, calcium signaling, and ECS pathways (CNR2, TRPV1 (17, 25, 56)). Polymorphisms in the TRPV1 gene, such as rs8065080, have emerged as potential shared susceptibility factors, critically modulating receptor function and autoimmune vulnerability (16, 17). Epistatic interactions between ECS-related polymorphisms (CNR2, TRPV1) and classical autoimmune susceptibility loci (PTPN22, CTLA4, FOXP3) further amplify autoimmune risk by synergistically impairing immune tolerance and ECS functionality (3, 8).

In addition to the genetic and ECS-related receptor vulnerabilities identified within affected endocrine and epithelial tissues, autoimmune disorders such as T1D, celiac disease, Hashimoto’s thyroiditis, Addison’s disease, and rheumatoid arthritis share well-defined immunological predispositions involving critical alterations in immune cell populations and inflammatory signaling pathways. These common immunological disturbances include compromised regulatory T-cell (Treg) differentiation and function, heightened pro-inflammatory T-helper 17 (Th17) responses (77), dysregulated macrophage polarization, and increased antigen-presenting cell (APC) activation (8, 29). Significantly, these immunological abnormalities demonstrate profound interactions with ECS signaling, reflected by altered endocannabinoid levels, specifically increased 2-arachidonoylglycerol (2-AG) and reduced anandamide (AEA), along with distinct receptor expression patterns of CB1, CB2, TRPV1, and TRPV2 within immune cells (16, 22, 25, 40).

A central shared immunological dysfunction across these autoimmune diseases involves impaired induction and stability of regulatory T-cells (Tregs). Tregs, crucial for maintaining peripheral immune tolerance and preventing autoimmune reactions, rely substantially on ECS signaling, particularly via CB2 receptor activation. CB2 receptor stimulation by AEA and, under physiological conditions, balanced 2-AG levels significantly promotes FOXP3 expression, enhances Treg differentiation, and reinforces immunosuppressive cytokine secretion (IL-10, TGF-β) (8, 25). Chronic ECS dysregulation characterized by sustained elevations of 2-AG results in profound CB2 receptor desensitization, diminishing Treg induction, suppressive function, and survival (16, 40). These immunological consequences are evident across T1D, CD, HT, AAD, and RA, where circulating Treg numbers and functionality are consistently compromised, correlating with increased autoimmune severity (29, 46).

Simultaneously, ECS dysfunction, especially CB2 receptor impairment due to chronic overstimulation, exacerbates pro-inflammatory T-helper 17 (Th17) cell responses, another critical autoimmune mediator common among these conditions. Reduced CB2-mediated signaling fails to restrain IL-17, IL-22, and IL-23 production, favoring Th17 cell differentiation, proliferation, and pathogenic activity (8, 25). Elevated Th17 cell populations and increased serum IL-17 and IL-23 concentrations are well-documented across autoimmune disorders such as T1D, CD, HT, AAD, and RA, highlighting a common ECS-mediated immunological imbalance potentiated by chronic 2-AG elevation and reduced CB2 signaling (16, 29, 46).

Further compounding these shared immunological predispositions is dysregulated macrophage polarization toward pro-inflammatory (M1) phenotypes driven by ECS dysfunction. Under physiological conditions, CB2 receptor activation by balanced endocannabinoid signaling promotes anti-inflammatory macrophage (M2) polarization (25, 40). Chronic 2-AG-driven receptor desensitization significantly disrupts this immunoregulatory function, increasing M1 polarization characterized by excessive secretion of TNF-α, IL-1β, and IL-6, intensifying tissue inflammation and autoimmune pathology (8, 29). Elevated pro-inflammatory macrophage populations and inflammatory cytokine profiles consistently accompany T1D, CD, HT, AAD, and RA, reflecting shared ECS-mediated immunological disturbances (16, 46).

Moreover, TRPV1 and TRPV2 receptor expression within immune cells, including macrophages, dendritic cells, and T-lymphocytes, represents an additional shared immunological vulnerability across these autoimmune diseases. TRPV channels mediate calcium-dependent immune cell activation, cytokine secretion, and apoptosis regulation (17, 24). Chronic receptor overstimulation, subsequent desensitization, and impaired calcium signaling due to elevated 2-AG contribute directly to dysregulated immune responses, APC hyperactivation, heightened pro-inflammatory cytokine production, and defective immune cell apoptosis, further amplifying autoimmune processes (22, 29). TRPV receptor dysfunction has been documented within immune cell populations in RA, HT, and CD (42, 50, 56), strongly suggesting similar mechanisms occur within T1D and AAD (25, 56, 59).

Finally, genetic susceptibility studies in autoimmune polyendocrine syndromes, particularly APS-2, consistently highlight polymorphisms in genes affecting ECS receptor function (CNR2, TRPV1) and immune regulatory genes (FOXP3, IL2RA, PTPN22) (22, 29, 42). Epistatic interactions between ECS-related polymorphisms and classical autoimmune susceptibility genes collectively exacerbate autoimmune vulnerability by synergistically impairing ECS-mediated immune regulation.

Taken together, these shared immunological disturbances—impaired Treg differentiation, heightened Th17 activity, dysregulated macrophage polarization, APC hyperactivation, and ECS receptor dysfunction—strongly support a unified ECS-mediated pathogenic mechanism underlying autoimmune vulnerability across T1D, CD, HT, AAD, and RA (8, 25, 29, 42, 56). This integrated immunological perspective further substantiates the ECBoM hypothesis, highlighting ECS restoration and microbiota-targeted interventions as promising therapeutic avenues for autoimmune diseases collectively (4, 22, 46).

Therefore, ECS dysfunction—exemplified by rapid TRPV1/2 receptor desensitization, compromised calcium signaling, and impaired CB2 immune regulation—represents a robust integrative pathogenic node consistently shared among T1D and frequently coexisting autoimmune disorders (8, 25, 29, 42, 56). Recognition of these common ECS-related vulnerabilities provides compelling evidence supporting the ECBoM hypothesis, suggesting that ECS dysfunction critically contributes to autoimmune pathogenesis across multiple disease contexts (22, 46).

Therapeutically, targeting ECS stabilization, microbiota restoration, and SCFA-mediated immunoregulation could offer substantial broad-spectrum benefits across these autoimmune conditions. Interventions such as selective TRPV modulators, CB2 receptor agonists, probiotics, dietary SCFA enrichment, and PPARγ activators could effectively interrupt autoimmune progression, preserving ECS functionality and restoring immune homeostasis (3, 8, 10, 25, 29, 40).

In summary, parallels in ECS dysfunction, TRPV receptor expression, calcium signaling disturbances, microbiota dysbiosis, and shared genetic susceptibility across T1D and commonly coexisting autoimmune disorders (CD, HT, AAD, RA) strongly substantiate the integrative ECBoM model. These findings underscore ECS’s central role in autoimmune pathogenesis, providing novel insights and potential therapeutic targets for managing autoimmune diseases collectively (8, 25, 29, 42, 56). However, it is important to note that much of the current evidence is derived from animal models and observational studies. While strong mechanistic overlaps exist, the extrapolation of these findings across disease contexts must be approached with caution. ECS dysfunction in T1D may exhibit unique characteristics not fully shared with other autoimmune diseases. Therefore, further targeted research is essential to validate the ECBoM hypothesis, confirm tissue-specific ECS alterations, and establish causality in human cohorts.

## Therapeutic potential: challenges & opportunities

The endocannabinoid system (ECS), acting as a dynamic interface between immune, metabolic, and neuronal networks, presents a compelling therapeutic target in the context of type 1 diabetes (T1D). As evidenced by the ECBoM model, ECS dysregulation—exemplified by chronic 2-arachidonoylglycerol (2-AG) elevation, receptor desensitization (especially of TRPV1), and downstream immune-metabolic dysfunction—constitutes a critical axis in T1D pathogenesis (1, 8, 17, 40). However, therapeutic manipulation of this complex system is fraught with both unique challenges and transformative opportunities (22, 25).

A primary challenge lies in the ligand promiscuity and bidirectional nature of endocannabinoid signaling. While CB2 activation confers robust anti-inflammatory and tolerogenic effects, CB1 stimulation—especially under chronic 2-AG excess—can exacerbate metabolic and inflammatory disturbances (7, 22). TRPV1, although protective under physiological stimulation, rapidly desensitizes under persistent activation, leading to loss of barrier integrity and mitochondrial dysfunction (17, 59). Moreover, ECS receptors are expressed not only on plasma membranes but also on mitochondrial membranes (mtCB1 and mtCB2), where they directly influence mitochondrial bioenergetics, apoptosis regulation, and reactive oxygen species (ROS) production (25, 40). This intracellular dimension of ECS signaling introduces further complexity, as chronic 2-AG diffusion into the inner mitochondrial membrane (IMM) can disrupt mitochondrial dynamics and compromise β-cell viability and immune homeostasis (1, 58).

Another promising direction lies in the use of dietary fatty acid precursors and lifestyle interventions to indirectly modulate ECS tone. Omega-3 fatty acids, for instance, can serve as precursors for endocannabinoid-like mediators (e.g., DHEA, EPEA) with anti-inflammatory properties and reduced receptor desensitization risk (4, 46). Preliminary data suggest these mediators may activate PPAR pathways without overstimulating CB1 or TRPV1 (22, 63). This opens the door to “nutritional ECS modulation,” particularly in individuals where pharmacological targeting may be premature or contraindicated. Combining these interventions with microbiota-supportive dietary patterns may yield synergistic effects, stabilizing both ECS and gut-derived immunoregulation (10, 11).

Despite these challenges, several therapeutic entry points emerge. One strategy involves rebalancing endocannabinoid tone by selectively inhibiting 2-AG biosynthesis or degradation pathways. For example, inhibition of diacylglycerol lipase (DAGL), the primary enzyme responsible for 2-AG synthesis, may attenuate receptor overstimulation and preserve CB2 and TRPV1 responsiveness (7, 14). Conversely, monoacylglycerol lipase (MAGL) inhibition, though anti-inflammatory in certain contexts, risks exacerbating 2-AG overload if not precisely titrated—highlighting the need for tissue-specific and temporally controlled interventions (7, 46).

Another avenue focuses on receptor-specific pharmacological modulation. CB2-selective agonists may restore peripheral immune tolerance, enhance regulatory T-cell (Treg) stability, and suppress pro-inflammatory cytokine release without inducing psychoactive effects (8, 25, 40). Simultaneously, partial TRPV1 agonists may stabilize channel function and prevent rapid desensitization, preserving epithelial barrier integrity and mitochondrial calcium signaling (17, 61). Additionally, novel ligands targeting mtCB1/2 receptors could regulate mitochondrial function in immune and β-cells, opening new frontiers in intracellular immunometabolic modulation (9, 23).

A complementary and potentially synergistic approach involves dietary and microbiota-directed strategies. Restoring short-chain fatty acid (SCFA) production—particularly butyrate—through prebiotic supplementation, fecal microbiota transplantation (FMT), or colonization with next-generation probiotics (e.g., *Faecalibacterium prausnitzii*) can significantly enhance PPARγ activation (4, 10, 78). This, in turn, strengthens Treg differentiation, tight junction integrity, and anti-inflammatory cytokine profiles, indirectly stabilizing ECS signaling via the gut–immune–endocannabinoid axis (3, 8). Enhancing SCFA–PPARγ–ECS crosstalk may prove especially beneficial in early-stage or pre-symptomatic individuals with elevated genetic risk (33, 34).

Nevertheless, personalized ECS-targeted therapy remains a formidable challenge. The ECS exhibits high interindividual variability, influenced by genetic polymorphisms in ECS-related enzymes and receptors, microbiota composition, and environmental factors (35, 36, 41). Thus, successful therapeutic intervention will likely require precision medicine frameworks, integrating genetic, microbial, metabolomic, and ECS receptor expression profiles to tailor intervention strategies (3, 46).

Furthermore, the psychoactivity of CB1 ligands and the systemic immunomodulatory effects of ECS-targeted therapies raise regulatory and ethical concerns, particularly in pediatric populations (7, 40). Strategies to bypass these concerns include developing non-psychoactive ECS modulators, optimizing targeted delivery systems (e.g., nanoparticle-based gut-restricted formulations), or focusing on peripheral CB2- and TRPV1-centric pathways (17, 25, 29).

In conclusion, while ECS dysregulation presents considerable therapeutic challenges in the context of T1D, it simultaneously unveils unprecedented opportunities for targeted immune and metabolic reprogramming (1, 46). Future research must strive to refine ECS-targeted interventions, prioritize safety and tissue specificity, and integrate ECS-modulating strategies with microbiota restoration and immunometabolic suport (3, 4). Through this integrative approach, it may become possible not only to delay or halt autoimmune progression but also to restore a measure of immune tolerance and metabolic stability in individuals at risk for, or already diagnosed with, type 1 diabetes.

## Future directions and experimental validation

In light of the central role played by the endocannabinoid system (ECS) in immune homeostasis, gut permeability, and inflammatory regulation (4, 8), further studies are warranted to investigate whether distinct biochemical signatures involving endocannabinoids and their associated immunometabolic markers can serve as predictors or modulators of autoimmune progression in Type 1 Diabetes (T1D) (3, 28). This design will also allow us to test the hypothesis that ECS dysregulation precedes measurable metabolic dysfunction, acting as an early indicator rather than a downstream consequence of β-cell loss (1, 20).

We propose a structured pilot study focused on the simultaneous quantification of circulating endocannabinoids—namely anandamide (AEA) and 2-arachidonoylglycerol (2-AG)—alongside key immunometabolic and gastrointestinal biomarkers in individuals with established T1D and in a comparative group of at-risk individuals, such as first-degree relatives of patients who test positive for diabetes-related autoantibodies (1). The aim is to explore whether dysregulation in ECS components correlates with measures of glycemic control (HbA1c, C-peptide), systemic inflammation (e.g., IL-6, TNF-α, IL-10, CRP) (8, 25), gut barrier integrity (zonulin, claudin-1, occludin), ionomic balance (Na^+^, K^+^, Ca²^+^, Mg²^+^, Cl^-^), and lipid metabolism (HDL, LDL, triglycerides, total cholesterol) (22, 26).

Given the mounting evidence linking intestinal dysbiosis and short-chain fatty acid (SCFA) depletion with ECS dysfunction, fecal samples will be analyzed for SCFA profiles, particularly levels of butyrate, propionate, and acetate, to establish potential associations between microbial metabolites and systemic ECS status (3, 10, 26). Moreover, assessment of zonulin and related markers of tight junction integrity will allow for the correlation of gut permeability with circulating inflammatory mediators and endocannabinoid tone (11, 13, 47).

As exploratory endpoints, the expression of CB1 and CB2 receptors at the mRNA level in peripheral blood mononuclear cells may be investigated, offering insights into receptor regulation under chronic inflammatory stress (23, 25, 56). This would allow the identification of receptor expression profiles potentially predictive of disease stage or progression.

To elucidate the temporal dynamics of ECS breakdown, longitudinal cohort studies are necessary, particularly in individuals at elevated risk for T1D but not yet diagnosed. Serial assessments of endocannabinoid levels, inflammatory cytokines, SCFA concentrations, and gut permeability markers over time would help determine whether specific ECS-related alterations precede the clinical onset of autoimmunity (4, 12, 19, 27). Likewise, follow-up studies in long-duration T1D patients may provide valuable information about ECS compensatory exhaustion, helping to delineate the tipping point beyond which immunometabolic resilience is lost (7, 46). Identifying this “immunological breaking point” may prove essential for designing timely and individualized preventive interventions.

The proposed pilot design includes cross-sectional profiling in a total of 70 individuals (50 with T1D and 20 at-risk), using multi-analyte platforms such as LC-MS/MS for endocannabinoid quantification and multiplex ELISA or Luminex for cytokine profiling (23, 25). Stool samples will be analyzed via gas chromatography for SCFA quantification, and RT-qPCR may be employed to assess cannabinoid receptor gene expression (8, 42). Blood samples will be collected in accordance with safe clinical limits for non-therapeutic studies, ensuring participant safety while allowing for the robust measurement of proposed biomarkers.

We envision that integrating ECS-specific biomarkers—such as 2-AG and AEA levels—with immunological and gut-derived parameters will not only refine our understanding of ECS involvement in T1D pathogenesis but may also unveil predictive signatures useful for early identification of at-risk individuals (8, 22, 28). Furthermore, the results from this pilot study could lay the foundation for the development of a broader ECS biomarker panel (ECBoM) capable of informing future preventive or therapeutic strategies targeting immunometabolic regulation in autoimmune diabetes (23, 25).

Ultimately, by focusing on non-interventional biomarker profiling in well-defined and longitudinally monitored cohorts, this research aims to bridge the translational gap between molecular ECS dysfunction and clinical T1D progression (3, 4, 25), opening new avenues for risk stratification, preventive screening, and personalized immunometabolic assessment (12, 28).

## Conclusions

The model presented here positions the endocannabinoidome–microbiota axis (ECBoM) as a central integrative node in the pathogenesis of type 1 diabetes (T1D). It proposes a stepwise mechanistic cascade in which gut dysbiosis initiates ECS hyperactivation, leading to receptor desensitization—particularly of TRPV1—and subsequent failure of key immunoregulatory and metabolic processes across intestinal, endothelial, and pancreatic tissues (4, 12, 25, 28).

This hypothesis synthesizes current insights from immunology, microbiology, and cannabinoid signaling into a unifying pathophysiological sequence. The ECS emerges not only as a modulator of immune responses but also as a buffering system whose failure may be decisive in triggering the autoimmune attack on pancreatic β-cells (8, 46). Notably, the desensitization of ECS receptors, especially TRPV1, under chronic 2-AG exposure appears to be a critical inflection point, stripping the host of essential protective mechanisms (17, 22, 25).

Importantly, models of ECS or microbiota dysfunction already exist independently, but the integration of both into a single coherent cascade opens entirely new perspectives on T1D pathogenesis (3, 4, 12). However, validating this model requires ambitious, multidimensional studies that combine multi-omics profiling, endocannabinoid quantification, inflammatory and barrier markers, and longitudinal follow-up of genetically at-risk individuals (30, 33, 34). These investigations are complex and resource-intensive, but they offer a paradigm shift in how we conceptualize autoimmune diabetes.

Ultimately, better understanding of ECS-microbiota crosstalk may enable the development of predictive biomarkers, early interventions, and novel immunometabolic therapies tailored to ECS tone and microbiota status (3, 12, 26). By shifting the focus from downstream immune destruction to upstream homeostatic failure, this approach provides a fresh therapeutic outlook on a disease long considered irreversible and unpreventable (8, 31).

## Data Availability

The original contributions presented in the study are included in the article/**Supplementary Material**. Further inquiries can be directed to the corresponding author.
