# Supplementary material for: Reframing type 1 diabetes through the endocannabinoidome-microbiota axis: a systems biology perspective
Source: Front Endocrinol (Lausanne). 2025 May 29;16:1576419. doi: 10.3389/fendo.2025.1576419 (PMC12158731; doi:10.3389/fendo.2025.1576419)
Supplement: Supplementary file 1 [file DataSheet1.pdf]

Pilot Study Protocol: Immunometabolic and Endocannabinoid Profiling in Type 1 Diabetes and At-Risk Individuals

Study Objectives

This pilot study aims to explore correlations between circulating endocannabinoid levels (AEA, 2-AG) and:

- Glycemic control (HbA1c, C-peptide)
- Inflammatory markers (CRP, IL-6, TNF-α, IL-10)
- Gut barrier integrity (zonulin, claudin-1, occludin)
- Fecal SCFA profiles (butyrate, acetate, propionate)
- Autoimmunity (GAD65 antibodies)
- Ion balance (ionogram)
- Extended lipid profile (HDL, LDL, TG, total cholesterol)
- CB1 and CB2 receptor mRNA expression (optional, see below)

This study will also investigate whether patterns in these biomarkers can distinguish between individuals with T1D and at-risk individuals (e.g., first-degree relatives with autoantibodies).

Study Design

- **Design:** Cross-sectional observational pilot study
- **Groups:**
  - 50 individuals diagnosed with Type 1 Diabetes (duration >1 year)
  - 20 at-risk individuals (positive for autoantibodies or first-degree relatives)
- **Age range:** 18–45
- **Exclusion criteria:** Chronic inflammatory diseases, active infections, recent antibiotics, immunosuppressants, cannabis use

---

Biosample Collection and Volume

Blood Sample Collection

Total: **~45 ml** per participant, collected in multiple tubes during one session

Tube Type	Purpose	Volume per Tube	Tubes
EDTA (purple cap)	Plasma for AEA, 2-AG, cytokines	6 ml	2
Serum (red or yellow cap)	HbA1c, lipid panel, ionogram, GAD65, C-peptide	6 ml	3

Tube Type	Purpose	Volume per Tube	Tubes
PAXgene RNA tube	CB1 and CB2 mRNA expression (if included)	2.5 ml	1
Heparin (green cap)	Backup for cytokines or additional metabolic panels	6 ml	1

Optional RNA expression module adds ~2.5 ml (1 PAXgene tube). This keeps the total below **50 ml**, a standard upper limit for adult participants in single-day, non-therapeutic blood sampling protocols.

Stool Sample Collection

- One fresh stool sample in sterile container
- Divided for:
  - SCFA profiling (gas chromatography)
  - Gut permeability biomarkers (zonulin, calprotectin, etc.)
  - Microbiota analysis (optional)

Methods

Category	Analytes	Methods
**Endocannabinoids**	AEA, 2-AG	LC-MS/MS
**Glycemic Control**	HbA1c, C-peptide	Immunoassay
**Inflammatory Markers**	IL-6, TNF-α, IL-10, CRP	Multiplex ELISA / Luminex
**Ionogram**	Na^+, K^+, Ca^{2+}, Mg^{2+}, Cl^-	Ion-selective electrodes
**Lipid Panel**	LDL, HDL, TG, TC	Standard clinical chemistry
**Autoimmunity**	GAD65 antibodies	ELISA
**Gut Barrier**	Zonulin, Occludin, Claudin-1	ELISA
**Fecal SCFAs**	Butyrate, acetate, propionate	GC-MS
**CB1/CB2 Expression** *(Optional)*	mRNA from blood	RT-qPCR (after RNA extraction from PAXgene tube)

Statistical Analysis

- **Primary correlations:** Spearman/Pearson coefficients between AEA/2-AG and HbA1c, C-peptide
- **Secondary analyses:** Group comparisons (T1D vs at-risk) using t-tests or Mann-Whitney U-tests
- **Multivariate modeling:** Stepwise regression or PCA to assess predictive value of EC + immune + metabolic panels

Ethics and Safety

- All participants will provide written informed consent
- Ethical approval will be obtained from a relevant institutional board
- The volume of blood collection is within safe limits for adults
- Data will be anonymized and handled in compliance with GDPR
